# Role of Hepatic Stellate Cells in the Early Phase of Liver Regeneration in Rat: Formation of Tight Adhesion to Parenchymal Cells

**DOI:** 10.1186/1476-5926-2-S1-S29

**Published:** 2004-01-14

**Authors:** Ayako Mabuchi, Ian Mullaney, Philip Sheard, Paul Hessian, Arthur Zimmermann, Haruki Senoo, Antony M Wheatley

**Affiliations:** 1 Department of Physiology, University of Otago, Dunedin, New Zealand; 2 Department of Pharmacology, University of Otago, Dunedin, New Zealand; 3 Department of Pathology, University of Berne, Switzerland; 4 Department of Microbiology and Immunology, Nippon Medical School, Tokyo, Japan; 5 Department of Anatomy, Akita University School of Medicine, Akita, Japan

## Abstract

We investigated activation mechanisms of hepatic stellate cells (HSCs) that are known to play pivotal roles in the regeneration process after 70% partial hepatectomy (PHx). Parenchymal liver cells (PLCs) and non-parenchymal cells (NPLCs) were isolated and purified from the regenerating livers at 1, 3, 7, 14 days after PHx. Each liver cell fraction was stained by immunocytochemistry using an anti-desmin antibody as a marker for HSCs, anti-alpha-smooth muscle actin (alpha-SMA) as a marker for activated HSCs, and 5-bromo-2'-deoxyuridine (BrdU) for detection of proliferating cells. Tissue sections from regenerating livers were also analyzed by immunohistochemistry and compared with the results obtained for isolated cell fractions. One and 3 days after PHx, PLC-enriched fraction contained HSCs adhered to PLCs. The HSCs adhered to PLCs were double positive for BrdU and alpha-SMA, and formed clusters suggesting that these HSCs were activated. However, HSC-enriched fraction contained HSCs not adhered PLCs showed positive staining for anti-desmin antibody but negative for anti-alpha-SMA antibody. These results suggest that HSCs are activated by adhering to PLCs during the early phase of hepatic regeneration.

## Introduction

The liver regenerates in size and function 7 to 14 days after 70% partial hepatectomy in rodents [1]. Recent reports demonstrated that not only proliferation of Parenchymal liver cells (PLCs) but also activation of sinusoidal liver cells, namely, Kupffer cells, hepatic lymphocytes, sinusoidal endothelial cells, pit cells, stem cells and HSCs are involved in the regeneration process through cell-cell interaction and cytokine networks [2]. The activated hepatic stellate cells (HSCs) proliferate vigorously, lose vitamin A and synthesize a large quantity of extracellular matrix. The morphology of these cells also changes from the star-shaped stellate cells to that of fibroblasts or myofibroblasts [3]. However, the molecular and cellular mechanisms of this process, especially, the roles of cell-cell interaction between PLCs and HSCs in the HSC activation remain unknown. In the present study, we isolated and purified HSCs and PLCs from regenerating liver after PHx in rats and investigated mechanisms of HSC activation from a viewpoint of adhesion between PLCs and HSCs *in vivo *and *in vitro*.

## Materials and Methods

### Animals and Partial Hepatectomy (PHx)

Female Lewis rats (200–250 g body weight) were used. Under ether anesthesia, rats were subjected to PHx using the method described by Higgins and Anderson [4] with minor modifications.

### Isolation of PLC- and HSC-enriched Fractions

Isolation and enrichment methods for PLCs and HSCs were a modification of the previously described isolation method for PLCs [5] and HSCs [6]. Briefly, the liver was perfused with Ca^2+^, Mg^2+^-free HBSS containing 0.05% collagenase at 37 degrees C. Then the liver was removed, cut into small pieces, and incubated in the same solution at 37 degrees C for 30 minutes. PLCs were separated from the non-parenchymal cells (NPLCs) by low-speed centrifugation. After washing with cold HBSS, the PLC-enriched fraction was obtained. HSCs were isolated from the NPLC-enriched fraction by 8.2% Nycodenz density gradient centrifugation. The HSCs-enriched fraction was obtained from an upper whitish layer.

### Immunohistochemistry

Indirect immunohistochemical examination of desmin and alpha-smooth muscle actin (alpha-SMA) was performed on formalin-fixed, and paraffin-embedded sections of rat liver.

### 5-bromo-2'-deoxyuridine (BrdU) Labeling for Proliferation Assay

BrdU (50 mg/kg body weight) was given to rats by an intraperitoneal injection 3 days after PHx. One hour later, the rats were used for isolation of liver cells.

### Immunocytochemistry of BrdU, Desmin and alpha-SMA

Each liver cell fraction freshly isolated from normal or PHx rats was re-suspended in PBS and adhered to microscope slides using a cytospin. Double immunocytochemical staining of desmin and BrdU was performed to demonstrate proliferating HSCs, while activating HSCs were shown by double immunocytochemical staining of desmin and alpha-SMA. Slides were observed under a fluorescence microscope and digitally photographed.

## Results

### Immunohistochemistry

To investigate the behavior of HSCs after PHx, we observed chronologically the regenerating liver by desmin and alpha-SMA immunohistochemistry and analyzed the activation of HSCs (data not shown). In summary, there was a clear increase of HSCs starting on day 1 which peaked on day 3 and declined again by day 7. HSC activation on day 14 was not different from day 0.

### HSCs "Contamination" in PLC-enriched Fraction

**Figure 1:**
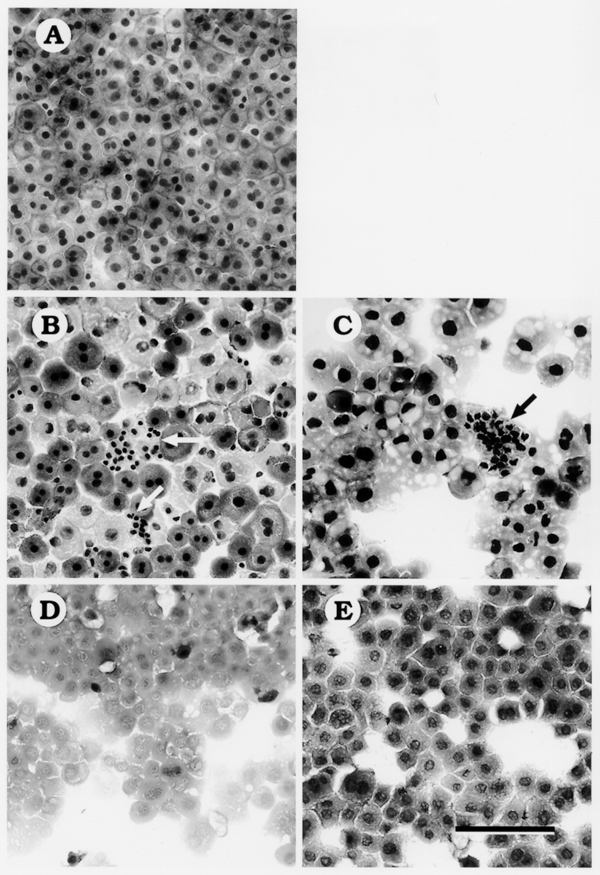
Giemsa staining of isolated PLC-enriched fraction. PLC-enriched fractions from normal (A), and PHx rats at 1 day (B), 3 days (C), 7 days (D) and 14 days (E) after PHx. Contaminating NPLCs (arrows) can be seen abutting or within regenerating PLC clusters. Bar = 100 –m.

**Figure 2:**
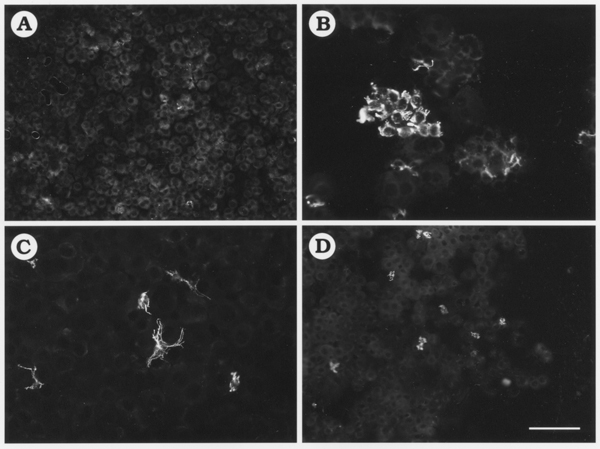
Immunodetection of desmin positive HSCs in PLC-enriched fraction. The cell fractions obtained from normal (A) and PHx rats at 1 day (B), 3 days (C), and 7 days (D) after PHx. Bar = 100 –m.

After PHx, we counted the number of NPLCs in the PLC-enriched fractions. We stained the PLC-enriched cell fraction with Giemsa, counted the number of NPLCs present in the fraction and calculated their percentage in the whole cell population. PLCs and NPLCs were readily discerned by cell and nucleus size (Fig. 1). In the PLC-enriched fraction obtained from normal rat liver, the percentage of NPLCs was 3%, and this increased to 27 and 20% at 1 and 3 days after PHx, respectively (data not shown). To identify HSCs in those NPLCs "contaminating" the PLC-enriched cell fraction, the fraction analyzed by desmin immunocytochemistry (Fig 2). Many desmin-positive cells were found in the PLC-enriched fraction at 1 and 3 days after PHx. To investigate proliferation of HSCs in PLC-enriched fraction, we analyzed the cell fraction by double immunocytochemistry using the anti-desmin antibody and BrdU staining (data not shown). Three days after PHx, HSC proliferation coincided with cluster formation. In addition, BrdU and desmin were co-localized within the same cells indicating that these HSCs were proliferating.

### Activation of HSCs by Adhering to PLCs

Further, we identified activated HSCs by double immunocytochemistry using anti-desmin and anti-alpha-SMA antibodies (data not shown). "Contaminating" HSCs in the PLC-enriched fraction at 3 days after PHx showed a double-positive reaction against the antibodies (data not shown). However, when HSCs were isolated separately from the same animal on a Nycodenz density gradient, HSCs were desmin-positive but not alpha-SMA positive (data not shown). Thus, HSC activation was detected only when HSCs adhered to PLCs in the PLC-enriched fraction. Activation was not demonstrated when HSCs were isolated and further purified as an HSC fraction from NPLC-enriched fraction.

## Discussion

We demonstrated previously that PLCs synthesize and release various cytokines such as GM-CSF [5], M-CSF, IL-1, and activate the hematopoietic and immune systems in the liver after PHx. In previous reports we have proposed as a hypothesis that the liver functions as a hematolymphoid organ. In our present study, the morphology of HSCs surrounding PLCs in the PLC-enriched fraction was round and HSCs were desmin positive at 1 day after PHx. Three days after PHx, the morphology of HSCs changed to that of myofibroblast-like cells and HSCs were alpha-SMA positive. The PLC-enriched fractions at 7 or 14 days after PHx contained virtually no HSCs. These data indicate that regeneration of liver structure including hepatic sinusoidal architecture, involves transient cluster formation by HSCs in the early phase.
